# Supplementary material for: Predicting visual function by interpreting a neuronal wiring diagram
Source: Nature. 2024 Oct 2;634(8032):113–23. doi: 10.1038/s41586-024-07953-5 (PMC11446822; doi:10.1038/s41586-024-07953-5)
Supplement: Supplementary file 6 — CRF and ERF predictions for individual TmY4 and TmY9 cells. Analogous to Supplementary Data 3, but for TmY target types. Shown are the top four monosynaptic pathways, the strongest pathway passing through each of the top ten intermediary types (ranking from Extended Data Fig. 7), and the trisynaptic pathway Tm1–TmY–Dm3–TmY (see the section entitled Prediction of spatial normalization). [file 41586_2024_7953_MOESM6_ESM.zip › DataS4/TmY9qΓèÑ/720575940618149972.pdf]

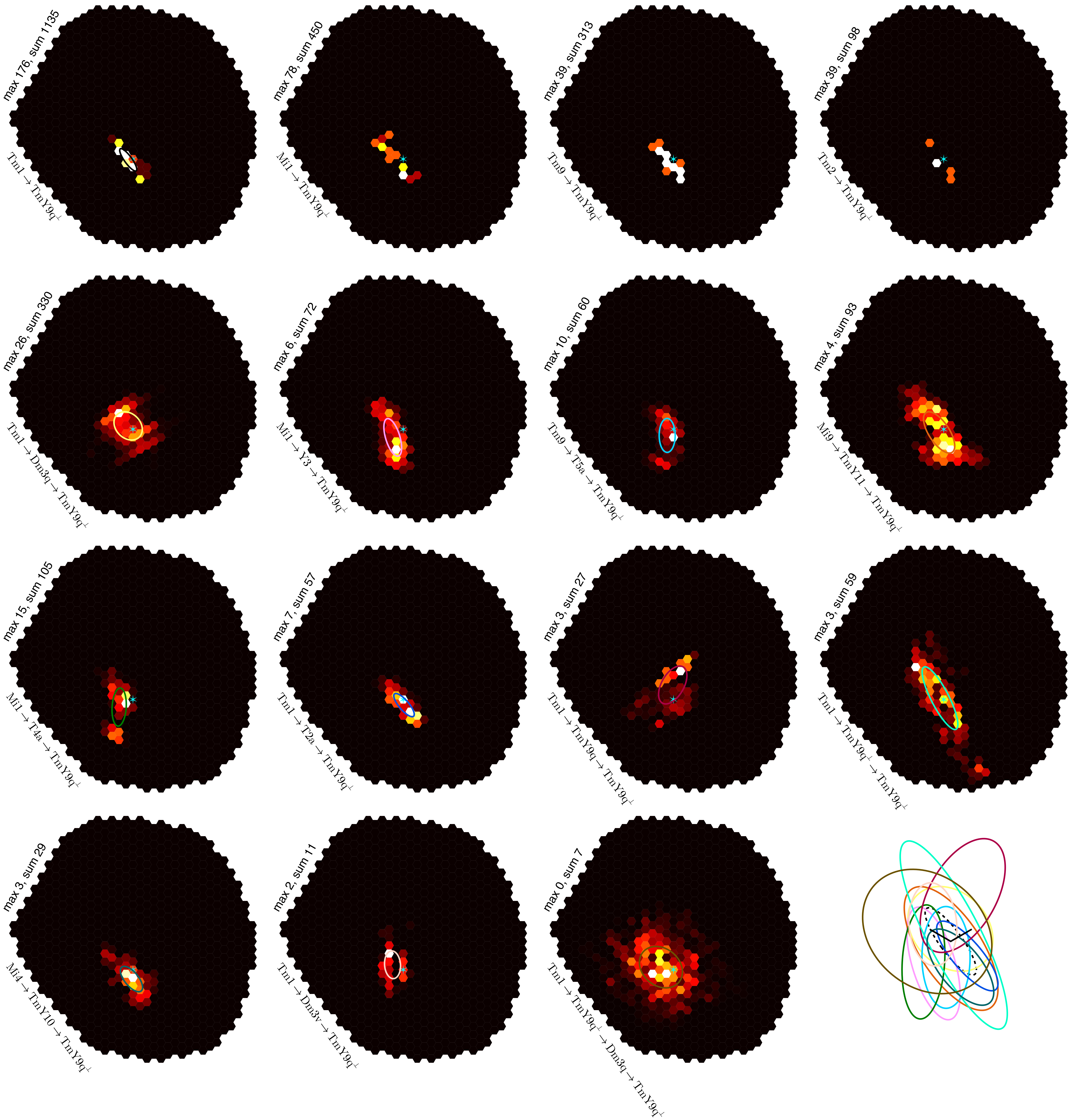
max 176, sum 1135
Tm1 → TmY9q⊥
max 78, sum 450
Mi1 → TmY9q⊥
max 39, sum 313
Tm9 → TmY9q⊥
max 39, sum 98
Tm2 → TmY9q⊥
max 26, sum 330
Tm1 → Dm3q → TmY9q⊥
max 6, sum 72
Mi1 → Y3 → TmY9q⊥
max 10, sum 60
Tm9 → T5a → TmY9q⊥
max 4, sum 93
Mi9 → TmY11 → TmY9q⊥
max 15, sum 105
Mi1 → T4a → TmY9q⊥
max 7, sum 57
Tm1 → T2a → TmY9q⊥
max 3, sum 27
Tm1 → TmY9q → TmY9q⊥
max 3, sum 59
Tm1 → TmY9q⊥ → TmY9q⊥
max 3, sum 29
Mi4 → TmY10 → TmY9q⊥
max 2, sum 11
Tm1 → Dm3v → TmY9q⊥
max 0, sum 7
Tm1 → TmY9q⊥ → Dm3q → TmY9q⊥
